# The Vacuolar H^+^ ATPase α3 Subunit Negatively Regulates Migration and Invasion of Human Pancreatic Ductal Adenocarcinoma Cells

**DOI:** 10.3390/cells9020465

**Published:** 2020-02-28

**Authors:** Mette Flinck, Sofie Hagelund, Andrej Gorbatenko, Marc Severin, Elena Pedraz-Cuesta, Ivana Novak, Christian Stock, Stine Falsig Pedersen

**Affiliations:** 1 Section for Cell Biology and Physiology, Department of Biology, Faculty of Science, University of Copenhagen, DK-2100 Copenhagen, Denmark; mette.flinck@bio.ku.dk (M.F.); knr881@alumni.ku.dk (S.H.); trv586@alumni.ku.dk (M.S.); elenapedraz@gmail.com (E.P.-C.); inovak@bio.ku.dk (I.N.); 2 Department of Pathology, Icahn School of Medicine at Mount Sinai, New York, NY 10029, USA; andrej.gorbatenko@yahoo.com; 3 Department of Gastroentero-, Hepato- and Endocrinology, Hannover Medical School, D-30625 Hannover, Germany; Stock.Christian@mh-hannover.de

**Keywords:** PDAC, TCIRG1, ATP6V0a3, invasion, migration, matrix degradation, proliferation, pH-regulation, autophagy

## Abstract

Increased metabolic acid production and upregulation of net acid extrusion render pH homeostasis profoundly dysregulated in many cancers. Plasma membrane activity of vacuolar H^+^ ATPases (V-ATPases) has been implicated in acid extrusion and invasiveness of some cancers, yet often on the basis of unspecific inhibitors. Serving as a membrane anchor directing V-ATPase localization, the a subunit of the V0 domain of the V-ATPase (ATP6V0a1-4) is particularly interesting in this regard. Here, we map the regulation and roles of ATP6V0a3 in migration, invasion, and growth in pancreatic ductal adenocarcinoma (PDAC) cells. a3 mRNA and protein levels were upregulated in PDAC cell lines compared to non-cancer pancreatic epithelial cells. Under control conditions, a3 localization was mainly endo-/lysosomal, and its knockdown had no detectable effect on pH_i_ regulation after acid loading. V-ATPase inhibition, but not a3 knockdown, increased HIF-1α expression and decreased proliferation and autophagic flux under both starved and non-starved conditions, and spheroid growth of PDAC cells was also unaffected by a3 knockdown. Strikingly, a3 knockdown increased migration and transwell invasion of Panc-1 and BxPC-3 PDAC cells, and increased gelatin degradation in BxPC-3 cells yet decreased it in Panc-1 cells. We conclude that in these PDAC cells, a3 is upregulated and negatively regulates migration and invasion, likely in part via effects on extracellular matrix degradation.

## 1. Introduction

Pancreatic cancer, of which pancreatic ductal adenocarcinoma (PDAC) comprises about 90% of cases, is one of the deadliest cancers globally, with a 5-year survival rate of less than 10% and predicted to be the second leading cause of cancer-related death in the USA by 2030 [1,2]. Reliable biomarkers are lacking, and most cases are diagnosed so late that surgical treatment is unfeasible. The standard of care is chemotherapy in the form of gemcitabine plus nab-paclitaxel, or FOLFIRINOX (leucovorin, 5-fluorouracil, irinotecan, and oxaliplatin). However, response rates are low and treatments prolong life only for weeks to a few months [1]. Hence, novel diagnostic and treatment options are urgently needed.

Solid tumors exhibit metabolic changes and high proliferative rates, causing increased acid generation and making cancer cells dependent on the upregulation of net acid extrusion [3,4]. In conjunction with restricted diffusion, this renders the tumor microenvironment highly acidic, while intracellular pH (pH_i_) is normal or alkaline [4,5]. Collectively this favors cancer cell proliferation and survival and promotes motility and invasiveness [3,4,5]. Well-studied net acid extruding ion transporters that are often highly expressed in cancer cells include Na^+^/H^+^ exchanger 1 (NHE1), Na^+^,HCO_3_^−^ cotransporters (NBCs), and monocarboxylate transporters (MCTs) [4,5]. V-type H^+^-ATPases (V-ATPases) have also been studied in the context of cancer [6,7]. In contrast to NHE1, NBCs and MCTs, V-ATPases predominantly localize to endosomes and lysosomes, the Golgi apparatus, and other intracellular compartments [8,9,10,11,12,13] and are only found in the plasma membrane in specialized cell types and some cancer cells [6,14,15]. V-ATPases play a pivotal role in controlling the luminal pH of endosomes, lysosomes, and the Golgi apparatus [6,7,14,16]. Through this, as well as through acidification-independent scaffolding functions, they regulate endocytic trafficking, autophagy, macropinocytosis, lysosomal degradation, metabolism, protein glycosylation, and signaling pathways including notch-, Wnt-, and epidermal growth factor receptor (EGFR) signaling [6,7,14,15,16].

V-ATPases consist of a peripheral V_1_ section responsible for ATP hydrolysis and a membrane-integral V_0_ section, responsible for H^+^ translocation. V_1_ comprises subunits A–H, and V_0_ subunits a, d, e, c, c’´ and accessory subunit Ac45. The a subunit (ATP6V0a), of which there are four isoforms, a1–4, forms a hemichannel mediating H^+^ transport and a transmembrane anchor important for V-ATPase localization [6,7,14]. The isoform expression pattern of ATP6V0a is cell type specific. ATP6V0a3 (a3) is the predominant a isoform in osteoclasts, where it localizes the V-ATPase to the plasma membrane, and a3 mutations are responsible for inherited forms of osteopetrosis [17].

Several highly invasive breast cancer cells exhibit elevated plasma membrane V-ATPase expression, and their in vitro invasion was shown to be reduced by V-ATPase inhibitors bafilomycin and concanamycin A (ConA) in a manner proposed to involve plasma membrane-localized V-ATPases [18,19]. Similar findings were reported in melanoma cells [20], whereas in prostate cancer cells, invasiveness was inhibited by bafilomycin in the absence of detectable V-ATPase expression in the plasma membrane [9,10]. In breast cancer cells, a3 knockdown (KD) reduced invasiveness, yet only by at most ~25% [21,22], and a role for a4 rather than a3 in invasiveness was proposed in 4T1-12B breast cancer cells [23].

V-ATPase function is of particular interest in PDAC, given the reliance of this exceptionally aggressive cancer on nutrient scavenging, and increased lysosomal catabolism, processes critically dependent on V-ATPase activity [24,25,26]. In PDAC patient tissue, expression of V-ATPase subunits V1E [27] and V0c [28] was reported to correlate with cancer stage. Furthermore, a3 was detected in the plasma membrane of invasive PDAC cells [27,29]. However, V-ATPase inhibition did not consistently reduce invasion in the PDAC cell lines [27,29], and conversely to what would be expected for a stimulatory role in invasion, V-ATPase inhibition upregulated the activity of matrix metalloprotease-2 (MMP-2) [27], a major matrix-degrading MMP in pancreatic cancers [30]. In contrast, V-ATPase activity is important for the degradation of MT1-MMP (also known as MMP14), which is highly expressed in PDAC cells [31,32] and is a key regulator of invasion [33]. Thus, a clear link between a3 and PDAC growth and invasiveness is lacking.

The aim of this study is to characterize the regulation and roles of a3 in PDAC cells. We report that a3 was upregulated in PDAC cell lines compared to non-cancerous pancreatic epithelial cells. In PDAC cells, a3 localized mainly to an endosomal/lysosomal compartment and its knockdown had no detectable effect on pH_i_ regulation after an acid load. V-ATPase inhibition, but not a3 knockdown, decreased PDAC cell proliferation and autophagic flux. Notably, a3 KD increased migration and invasion of Panc-1 and BxPC-3 PDAC cells, and increased gelatin matrix degradation by BxPC-3 cells but decreased it in Panc-1 cells. Thus, in these cancer cells, a3 is upregulated and negatively regulates migration and invasiveness. No major roles of a3 in favoring PDAC development could be detected, although their existence in settings not studied here remains possible.

## 2. Methods

### 2.1. Reagents

Unless otherwise mentioned reagents were from Sigma-Aldrich (St. Louis, MO, USA) and were of the highest analytical grade. Antibodies against AMPK, phospho-Thr172-AMPK, LC3B, GAPDH, Golgin 97, Rab7, PARP, phospho-Ser780 pRb, and phospho-Ser15 p53 were obtained from Cell Signaling Technology (Danvers, MA, USA). Antibodies against V-ATPase B2 subunit, E-cadherin, p21, and LAMP1 were obtained from Santa Cruz Biotechnology (Santa Cruz, CA, USA), and antibody against β-actin was from Sigma-Aldrich. Antibodies against alpha-adaptin 2, MT1-MMP (MMP14) and p62 were from Abcam (Cambridge, UK). HIF-1α and p150^Glued^ antibodies were from BD Biosciences (San Jose, CA, USA). Antibodies against Giantin and HSP47 were from Enzo Lifesciences (Farmingdale, NY, USA). HRP-conjugated secondary antibodies for Western blotting (goat-anti-mouse (GAM) and goat-anti-rabbit (GAR)) were from DAKO (Glostrup, Denmark). Rhodamin phalloidin, Alexa Fluor 568 conjugated GAM, and Alexa Fluor 488 conjugated GAR secondary antibodies for immunofluorescence were from Invitrogen (Carlsbad, CA, USA). Antibody against Cortactin was from Merck (Darmstadt, Germany). Concanamycin A, Forskolin, and antibody against TCIRG1 (V-ATPase a3 subunit) were from Sigma-Aldrich (St. Louis, MO, USA).

### 2.2. Cell Culture and Treatments

BxPC-3 cells (American Type Culture Collection, ATCC) were cultured in RPMI-1640 medium (Gibco, #61870-010, 11 mM glucose) and Panc-1 cells (ATCC) in Dulbecco’s modified Eagle medium (DMEM, Gibco, #32430-027, 25 mM glucose), high glucose, all supplemented with Glutamax (Life Technologies, Camarillo, CA, USA), 10% fetal bovine serum (Gibco, #10 106-177), and 100 U/mL penicillin, 100 μg/mL streptomycin (Pen/Strep, Invitrogen, Carlsbad, CA #15140-148). For starvation conditions, cells were grown in DMEM (Gibco, #11966-027) or RPMI-1640 (Gibco, #11879020) media without glucose, 1% fetal bovine serum and 100 U/mL penicillin, 100 µg/mL streptomycin. Immortalized normal human pancreatic ductal epithelial (HPDE H6c7) cells were kindly provided by Dr M.-S. Tsao, Ontario Cancer Institute, Toronto, Canada [34,35] and cultured in kerantinocyte basal medium supplemented with epidermal growth factor and bovine pituitary extract. All cell cultures were maintained at 37 °C, 95% humidity and 5% CO2.

### 2.3. siRNA and Transfection

The pre-designed siRNAs against a3 and a negative control siRNA were from Origene (Rockville, MD, USA, #SR306986). Plasmids used were LAMP1-mCherry in pEGFP-N1 (GFP removed), a gift from Bin Liu, Danish Cancer Society Research Center, Denmark, and human TCIRG1 (a3) in pMA, a gift from Johan Richter, Lund University, Sweden. Both were verified by sequencing. Cells were transfected with the relevant siRNA (10 nM final concentration) or plasmid using Lipofectamine 2000 or -3000 (Life Technologies, Camarillo, CA, USA, #11668019), 48 h before start of the experiment. Then, 4 h after transfection, the medium was changed to either growth or starvation medium.

### 2.4. SDS-PAGE and Western Blotting

After treatments as indicated, cells were lysed in 95 °C SDS lysis buffer (1% SDS, 0.1 M Tris-HCl, 0.1 M NaVO_3_, pH 7.5) supplemented with Complete™ protease inhibitor mix (Roche Diagnostics GmbH, Germany, #11836153001). Lysates were homogenized by sonication (PowerMED, Portland, Maine), centrifuged (Micromax RF, Thermo) for 5 min at 20,000× *g* at 4 °C, and protein concentrations determined using DC Protein assay kit (BioRad, Hercules, CA, USA, #500-0113, #500-0114, #500-0115). Samples were equalized with ddH_2_O and NuPAGE LDS 4x sample buffer (5 mM Tris-Cl pH 6.8, 10% SDS, 1% bromophenol blue, 10% glycerol; Life Technologies, Carlsbad, CA, USA, #NP0007) and dithiothreitol added. Equal amounts of protein per lane were separated by SDS-PAGE, using Tris/glycine/SDS running buffer (BioRad, Hercules, CA, USA, #161-0732), precast Criterion 10% TGX gels (BioRad, Hercules, CA, USA, #567-1034 (18-wells) or #567-1035 (26-wells)), and BenchMark protein ladder (Life Technologies, Carlsbad, CA, USA, #10747-012). Proteins were transferred to Trans-Blot Turbo 0.2 μm nitrocellulose membranes (BioRad, Hercules, CA, USA, #170-4159). Membranes were Ponceau S stained (Sigma-Aldrich, St. Louis, MO, USA, #P7170-1L), blocked for 1 h at 37 °C in 5% nonfat dry milk in TBST (0.01 M Tris/HCl, 0.15 M NaCl, 0.1% Tween 20, pH 7.4), incubated with primary and secondary antibodies, and developed using ECL (Pierce™ ECL Western Blotting Substrate (Bio-Rad, Hercules, CA, USA, Cat. #1705061) or SignalFire (Cell Signaling, Danvers, MA, USA, #6883) and the Fusion Fx system (Vilber Lourmat, Marne-la-Vallé, France) for HRP-conjugated secondary antibodies. Densitometric analyses were carried out using UN-SCAN-IT 6.1 (Silk Scientific, Orem, Utah), or ImageJ software v1.52s.

### 2.5. Quantitative Real-Time PCR (qPCR)

Isolation of total RNA was performed using *NucleoSpin*® *RNA II* (Macherey-Nagel, Germany) according to the manufacturer’s instructions. RNA was reverse-transcribed using Superscript III Reverse Transcriptase (Invitrogen, Carlsbad, CA, USA, #18080044) and cDNA amplified by qPCR using SYBR Green (Roche, Basel, Switzerland, #04913914001) in an ABI7900 qPCR machine, in triplicate and using the steps: 95 °C for 10 min, 40 cycles of [95 °C for 30 s, 55 °C for 1 min, 72 °C for 30 s], 95 °C for 1 min. Primers were designed using NCBI/ Primer-BLAST (www.ncbi.nlm.nih.gov) and synthesized by Eurofins Genomics, Ebersberg, Germany (ATP6V0a1 and ATP6V0a2 and β-actin) or Invitrogen, Carlsbad, Ca, USA (ATP6V0a3, ATP6V1B2). Primer sequences: ATP6V0a1, sense 5′-GAGGAGGCAGACGAGTTTGA-3′; antisense 5′-CCGGTCCCGCTGTACAATTT-3′, ATP6V0a2, sense 5′-GGTTATCGCGCTCTTTGCAG-3′; antisense 5′-TTCTACCCAGTGGAGGCGTA-3′, ATP6V0a3, sense 5′-GTGAATGGCTGGAGCTCCGATGA-3′; antisense 5′-AGGCCTATGCGCATCACCATGG-3′ and ATP6V1B2, sense 5′- AGTCAGTCGGAACTACCTCTC-3′; antisense 5′-CATCCGGTAAGGTCAAATGGAC-3′; β-actin sense 5′-AGCGAGCATCCCCCAAAGTT-3′, antisense 5′-GGGCACGAAGGCTCATCATT-3′. mRNA levels were determined using the comparative threshold cycle (Ct) method, normalized to β-actin, and were expressed relative to that in HPDE cells or relative mock ctrl.

### 2.6. Immunofluorescence Imaging

Cells grown on glass coverslips were washed in ice-cold phosphate-buffered saline (PBS), fixed in 2% paraformaldehyde (Sigma, St. Louis, MO, USA, #47608) for 15 min at room temperature, washed in TBST (2 × 5 min), permeabilized for 5 min in 0.1% Triton x-100 (Sigma-Aldrich, St. Louis, MO, USA, #T8787) in TBST, blocked for 30 min in 5% BSA in TBST, and incubated at room temperature (RT) for 1.5 h or overnight at 4 °C with primary antibodies diluted in TBST + 1% BSA. The next day, preparations were washed in TBST + 1% BSA (3 × 5 min), and incubated for 1 h at room temperature with the relevant fluorophore-conjugated secondary antibodies diluted in TBST + 1% BSA. Finally, preparations were washed in TBST + 1% BSA for 3 × 5 min, of which the second wash contained 4’,6-diamidino-2-phenylindole (DAPI; Invitrogen, Carlsbad, CA, USA, #C10595) for nuclear staining. Coverslips were mounted in N-propyl-gallate antifade mounting media (Sigma, St. Louis, MO, USA #P-3130) on glass slides and sealed with nail polish. Cells were visualized using the 60X/1.35 Oil or 40X/1.0 NA objective of an Olympus BX63 or IX83 epifluorescence microscope. Z-stacks were deconvoluted in Olympus cellSens software using a constrained iterative algorithm. No or negligible labeling was seen in the absence of primary antibody. Overlays and brightness/contrast adjustment was carried out using ImageJ software. No other image adjustment was performed.

### 2.7. Gelatin Degradation Assay

Coverslips were coated with 60 °C preheated Oregon-green conjugated gelatin (Invitrogen, Carlsbad, CA, USA, #G13186) at 0.5 mg/mL in PBS + 2% sucrose, then gently aspirated with a soft vacuum source to form a thin uniform coat. Each coverslip was placed in a light-protected 12-well plate and left to air dry until the coating became whitish. Then, 1 mL of pre-chilled 0.5% glutaraldehyde solution (Sigma, St. Louis, MO, USA, #G6257) was added to each well and incubated for 15 min on ice. Coverslips were washed 3 times with PBS at RT, followed by addition of 1 mL freshly prepared sodium borohydride (5 mg/mL) (Sigma, St. Louis, MO, USA, #452882) solution to each well, incubation for 3 min, and wash in PBS. The desired number of coated coverslips was transferred into a new sterile 12-well plate, followed by seeding of cells to reach a confluence of 60% on the day of fixation. Cells were prepared for immunofluorescence analysis as above, using DAPI to visualize nuclei and Alexa Fluor 568–phalloidin to visualize F-actin (Invitrogen, Carlsbad, CA, USA, # R415).

### 2.8. Measurements of Intracellular pH (pH_i_)

Cells seeded on 15 mm glass coverslips were transfected with mock or a3 siRNA as above. After 48 h, cells were loaded for 30 min with 2 µM BCECF-AM (ThermoFischer Scientific, Waltham, MA, USA #B1150) in Ringer (in mM: NaCl 125, KCl 5, CaCl_2_ 1, MgCl_2_ 0.5, Na_2_HPO_4_ 1, glucose 11, HEPES 25), followed by two washes in this solution. Coverslips were mounted in the perfused chamber of an Imic2000 microscope equipped with a gravity flow perfusion system, and images acquired using a 40× oil immersion objective (Olympus, Tokyo, Japan), a PolychromeV monochromator light source (Till Photonics, Gräfelfing, Germany), appropriate Chroma filter set (Chroma Technology, Bellows Falls, VT, USA), and an Ixon 885 camera (Andor, Belfast, N. Ireland). Every 2 s, fluorescence signals were collected at 470–550 nm after excitation at 440 and 485 nm, using Till Photonics Live Acquisition software. Experiments were done at 37 °C, in the absence of CO_2_/HCO_3_^−^ to facilitate isolation of the contribution of V-ATPases. After achieving a stable baseline pH_i_, cells were exposed to 20 mM NH_4_Cl in Ringer for 10 min, followed by a ~3 min exposure to Na^+^-free saline (replaced by N-methyl-D-glucammonium (NMDG^+^)). Calibration was performed using the high-K/nigericin technique, essentially as in [36]. Intrinsic intracellular buffering capacity (β_i_) was calculated from the change in pH_i_ upon washout of NH_4_Cl under Na^+^-free conditions, using the equation β_i_ = Δ[NH_4_^+^]_i_/ΔpH_i_. [NH_4_^+^]_i_ was calculated using the Henderson–Hasselbalch equation and assuming a pKa of 8.9. Net Na^+^-and CO_2_/HCO_3_^−^-independent acid efflux (J) was calculated as the change in pH_i_ during the last min of the Na^+^-free phase, multiplied by β_i_, as: *J.* = dpH_i_/dt × β_i_.

### 2.9. Single Cell Migration Analysis

T-12.5 flasks were coated with a substratum composed of extracellular matrix-mimicking components including laminins (4.46%), fibronectin (4.46%), collagen IV (0.60%), collagen III (1.34%), collagen I (89.15%). The coating was let solidify overnight in an incubator at 37 °C. Transfected cells were thinly seeded at a density of 2 × 10^4^ cells per flask and allowed to adhere for 3–4 h at 37 °C, 5% CO_2_ prior to placing flasks in a heated chamber (37 °C) on the stage of an inverted microscope (Axio Vert.A1; 20× objective; Zeiss, Oberkochen, Germany) for recording. A representative field displaying at least eight cells was chosen for evaluation. Migration was monitored for 10 h with an ORCA C8484-05G02 video camera (Hamamatsu, Herrsching, Germany) controlled by HC-IMAGE-Live software (version 4.2.5; Hamamatsu). Images were taken at 10 min intervals and stored as stacks of TIF-files. Single cell migration was manually tracked and data extracted using ImageJ (http://rsb.info.nih.gov/ij/). Migration is represented by the movement of the nucleus during a defined time interval. Migration velocity (μm min^−1^) was calculated as the mean of the velocities for each time (10 min) interval, and is a measure of the average speed of movement during the experiment. Translocation was calculated as the mean distance between the position of the nucleus at the beginning and at the end of the experiment. The total path length was determined as the sum of the single distances covered within each time interval. A directionality index as measure of sustained, directed migration was calculated dividing the translocation (start-end distance) by the total path length. Thus, a directionality index of “1” would represent a straight linear forward movement.

### 2.10. Cell Invasion Analyses

Invasion was analyzed using 24-well 8 µm transwell BioCoat Chambers (BD Biosciences, Franklin Lakes, NJ, USA). Briefly, cells were starved in low serum media (1% FBS) supplemented with 0.1% BSA. After 24 h, cells were harvested and resuspended in serum-free medium. Then, 2.5 × 10^4^ cells/mL cells were seeded onto the growth factor reduced Matrigel invasion chambers (BD Biosciences #354483) to analyze invasion and, in parallel wells, onto cell culture inserts (BD Biosciences, #354578) to analyze migration. Medium containing 10% FBS was added to the lower chambers and chambers were incubated at 37 °C and 5% CO_2_ for 24 h. After washing with Gurr buffer, non-invading cells were gently removed using a cotton swab. Invasive cells located on the lower side of the chamber were fixed in ice cold absolute methanol for 30 min and stained with 30% Giemsa staining solution for 30 min. Membranes were washed 3× in Gurr buffer and air-dried. Stained membranes were cut out and placed on a glass slide. Ten random fields of view per membrane were imaged using the 40× objective of an Olympus Bx63 microscope and an Olympus D73 camera. Data are shown as the ratio of invaded/migrated cells over these 10 fields of view.

### 2.11. Proliferation Assay

Cells were seeded in 96-well plates in triplicates and at 0.3 × 10^3^ and 10^4^ cells per well for each condition. After 24 h, BrdU labeling reagent was added (Roche, Basel, Schwitzerland, #11647229001). After 4 h, cells were fixed and stained with BrdU antibody according to the manufacturer’s protocol (Roche, Basel, Schwitzerland, #11647229001). BrdU incorporation was measured on FLUOstar OPTIMA microplate reader (BMG Labtech, Ortenberg, Germany).

### 2.12. 3D spheroid Growth and CellTiter Glo Assay

First, 1000 Panc-1 or BxPC-3 cells were seeded per well in round bottomed, ultra-low attachment 96-well plates (Corning, NY, USA, # 7007) in 200 μL growth medium. Cells were subsequently spun down at 750 RCF for 15 min, and were grown for 9 days at 37 °C with 95% humidity and 5% CO_2_. Media for Panc-1 spheroids was supplemented with GelTrex LDEV-Free reduced growth factor basement membrane matrix (ThermoFisher Scientific, Waltham, MA, USA, #A14132029). Then, 100 μL medium was exchanged every second day. Light microscopic (Leica MZ16, Germany) images of the spheroids at 10× magnification were acquired on days 2, 4, 7, and 9. On day 9, 100 µL medium was replaced with 50 µL CellTiter-Glo® 3D Reagent (Promega, Madison, WI, USA, #G9683). The plates were shaken for 5 min, incubated for 25 min at room temperature and luminescence recorded using a FLUOStar Optima microplate reader (BMG Labtech, Ortenberg, Germany). 

### 2.13. Statistics

Unless otherwise specified, data are presented as mean ± S.E.M. of at least three independent experiments. Data analysis was performed using SPSS software and Graphpad Prism 6.0. Multiple groups were analyzed by one-way ANOVA with Dunnett’s post-hoc test as relevant; Student’s *t*-test was used for comparisons of datasets with only two groups; *p*-values of <0.05 were considered statistically significant.

## 3. Results

### 3.1. a3 is Upregulated in PDAC Cells and Mainly Localizes to Late Endosomes and Lysosomes

**Figure 1:**
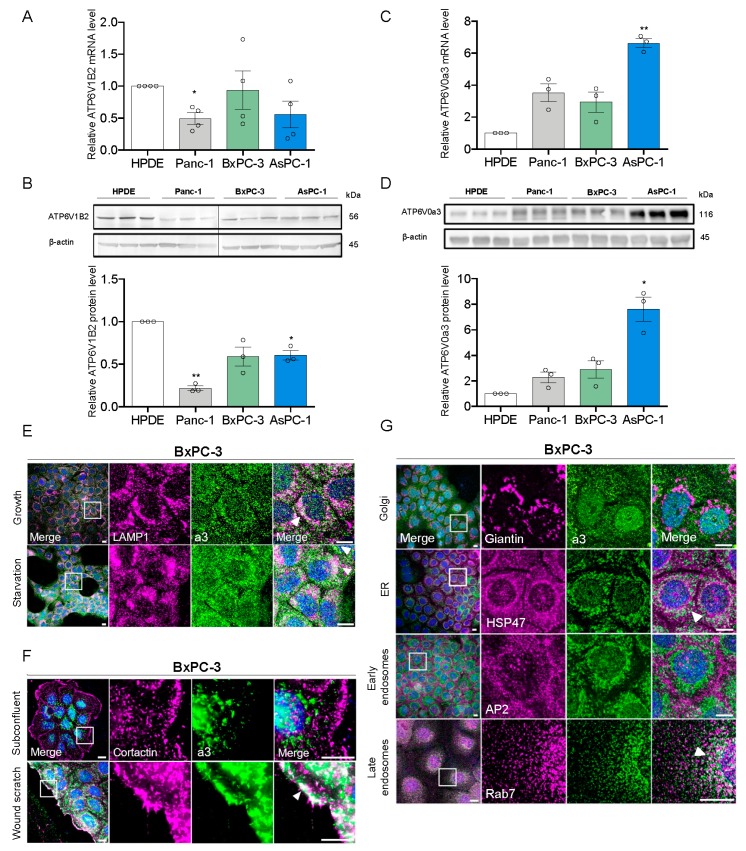
a3 is upregulated in PDAC cells and mainly localizes to late endosomes and lysosomes. (**A**,**C**) Relative mRNA levels of ATP6V1B2 and ATP6V0a3 in PDAC cells assessed by qRT-PCR. Data is normalized to β-actin, expressed relative to that in HPDE cells, and shown as mean with S.E.M. error bars, of 3 (ATP6V0a3) to 4 (ATP6V1B2) independent experiments per cell line. * *p* < 0.05 and ** *p* < 0.01: Significantly different from the level in HPDE cells, using one-way ANOVA and Dunnett’s post hoc test. (**B**,**D**) Protein levels of ATP6V1B2 and ATP6V0a3 in PDAC cells, assayed by Western blotting. Upper panels show representative blots, and lower panels show quantification normalized to β-actin (loading ctrl.) and the level in HPDE cells. Data is shown as mean with S.E.M. error bars, of 3 independent experiments per cell line. * *p* < 0.05 and ** *p* < 0.01: Significantly different from the level in HPDE cells, using one-way ANOVA and Dunnett’s *post hoc* test. (**E**,**F**) BxPC-3 cells cultured under growth, starvation, subconfluent or wound scratch conditions as indicated were subjected to immunofluorescence analysis for a3 (green) and LAMP1 or cortactin (magenta). Nuclei were stained with DAPI. Scalebar = 10 µm. White arrowheads indicate colocalization. Images represent 2–5 independent experiments. (**G**) Immunofluorescence analysis of a3 (green) and either Giantin (Golgi), HSP47 (ER), AP2 (early endosomes), or Rab7 (late endosomes) (all magenta) in BxPC-3 cells. Nuclei were stained with DAPI. Scalebar = 10 µm. White arrowheads indicate colocalization. Images are representative of 2–3 independent experiments.

To study the potential roles of a3 in PDAC cells we first determined mRNA- and protein levels of this subunit compared to B2 (the only B subunit isoform in somatic cells and thus a proxy for total V-ATPase expression) in non-cancerous, immortalized human pancreatic epithelial cells (HPDE cells) and three human PDAC cell lines: Panc-1, BxPC-3, and AsPC-1. Both mRNA and protein levels of B2 were unaltered or slightly reduced in PDAC cells compared to HPDE cells (Figure 1A,B). In contrast, a3 mRNA and protein levels were increased in all PDAC cells, with 6- to 8-fold upregulation in AsPC-1 compared to that in HPDE cells (Figure 1C,D). BxPC-3 and Panc-1 cells were selected for further analysis as examples of PDAC cells expressing wild type (BxPC-3) or constitutively active (Panc-1) KRAS [37], which was recently shown to be important for V-ATPase function in PDAC cells [15]. 

In confluent BxPC-3 cells (Figure 1E, top panel), the majority of a3 labeling was intracellular and punctate, and overlapped substantially with staining for LAMP1 to visualize lysosomes (white arrowheads). A similar pattern was seen in Panc-1 cells (Figure 1A, top panel) cells and this colocalization was also observed when a3 and LAMP1 were overexpressed (Figure S1B,C). In BxPC-3 cells, there was partial colocalization of a3 with Rab7 (late endosomes), and to a lesser extent with heat shock protein (HSP)47 (endoplasmic reticulum), but negligible colocalization with Giantin (cis- and medial Golgi) and AP2 (early endosomes) (Figure 1G). To address if a3 membrane localization was dependent on confluence or nutrient status, we determined a3 localization (i) following glucose- and serum starvation (Figure 1E, Figure S1A, lower panels), (ii) in subconfluent cultures (Figure 1F, Figure S1D–F), and (iii) after introduction of a wound scratch (Figure 1F; Figure S1D). Colocalization between a3 and LAMP1 was not altered by starvation (Figure 1E, Figure S1A, lower panels). There was no detectable colocalization of a3 with cortactin (plasma membrane) under subconfluent conditions (Figure 1F), yet after a wound scratch, a distinct relocalization of a3 to the plasma membrane was seen in BxPC-3 cells (Figure 1F). In Panc-1 cells, no a3 membrane localization was detected under these conditions (Figure S1D). In contrast, in subconfluent Panc-1 cells, but not in subconfluent BxPC-3 cells, a3 localized in a highly perinuclear pattern surrounding the Golgi apparatus (Figure S1E,F). The same pattern was seen for LAMP1 (Figure S1E,F), consistent with translocation of lysosomes to the perinuclear region (see Discussion).

These results show that a3 expression is increased in PDAC cells compared to immortalized pancreatic epithelial cells, and that a3 localizes predominantly to late endosomes and lysosomes in Panc-1 and BxPC-3 cells.

### 3.2. a3 Is Not. Upregulated by Starvation and Does Not. Contribute to pH_i_ Recovery After Acidification

**Figure 2:**
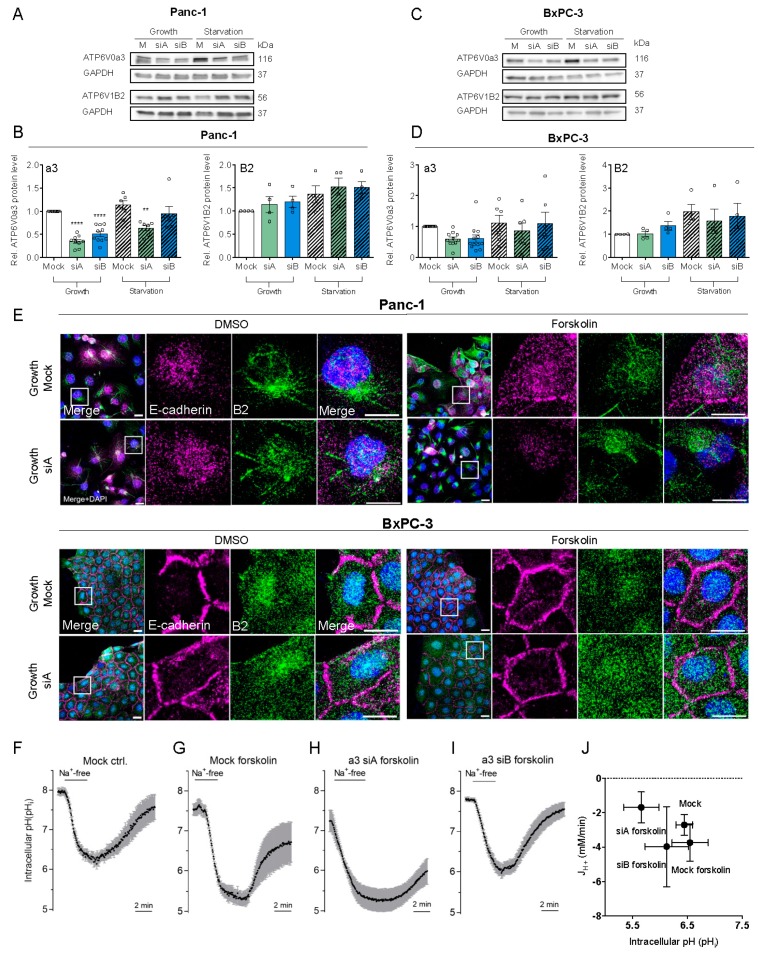
a3 is not upregulated in starvation and its KD does not inhibit pH_i_ recovery. Panc-1 and BxPC-3 cells were transfected with mock siRNA or 2 different siRNAs targeting a3 and grown under growth or starvation conditions for 48 h, lysed and subjected to Western blotting for a3 and B2. Upper panels (**A**,**C**) show representative blots and lower panels (**B**,**D**) show densitometric quantification normalized to GAPDH (loading ctrl.) and the level in respective mock controls. Data is shown as mean with S.E.M. error bars, of 3 to 12 independent experiments per cell line and siRNA. ** *p* < 0.01 and **** *p* < 0.0001: Significantly different from the level in control conditions (mock siRNA), using one-way ANOVA and Dunnett’s post hoc test. Rel. = relative. (**E**) Panc-1 and BxPC-3 were transfected with Mock siRNA or 2 different siRNAs targeting a3, and were further treated with 10 μM forskolin or vehicle ctrl. (DMSO). Cells were grown for 48 h, and subjected to immunofluorescence analysis for B2 (green) and E-cadherin (magenta). Nuclei were stained with DAPI. Scalebar = 20 µm. (**F**–**J**) BxPC-3 cells were transfected with mock siRNA (**F**,**G**) or siRNA-A or -B against a3 (**H**,**I**) as indicated, and 48 h later, were subjected to live imaging of pH_i_ using BCECF-AM under CO_2_/HCO_3_^−^ free conditions. Where indicated, forskolin (10 µM) was present during the 30 min loading of with BCECF. The horizontal bar indicates the switch from standard HEPES-buffered Na^+^-containing Ringer with 20 mM NH_4_Cl to induce the acid load, to NMDG-Cl Ringer to observe Na^+^-independent pH_i_ recovery. (**J**) The Na^+^-and CO_2_/HCO_3_^−^ independent net acid efflux over time was calculated as the change in pH_i_ during the last minute of the Na^+^-free phase, multiplied by β_i_ to obtain the flux, J_H+_, and plotted against the mean pH_i_ during the minute of the measurement. BCECF ratios were calibrated to pH_i_ using the high K^+^/nigericin technique. Data shown are representative of (**F**–**I**) or summarized (**J**) from 3 independent experiments. Error bars show SD of measurements on multiple cells in the same experiment (**F**–**I**) or S.E.M. of 3 independent experiments.

In mammalian cells, V-ATPase assembly and function are increased by glucose starvation [38]. We therefore tested the effect of glucose and serum starvation (0 mM glucose, 1% serum) on a3 and V-ATPase expression. Protein (Figure 2A–D) and mRNA (Figure S2E,I) expression of B2 tended to be increased by starvation and were largely unaltered by a3 KD, which also had no detectable effect on a1 and a2 mRNA levels (Figure S2B–I), as previously reported in osteoclasts [39]. In complete medium, 50–60% knockdown of a3 was obtained in both Panc-1 and BxPC3 cells (Figure 2A–D). Under starvation conditions, only about 25% a3 KD could be obtained at the protein level (Figure 2A–D) while it was about 50% at the mRNA level (Figure S2D,H), precluding further analysis of the effect of a3 KD under starvation conditions. To stimulate V-ATPase membrane localization, we used forskolin to increase cAMP, which promotes V-ATPase membrane insertion in some cell types [40]. B2 localization was not detectably affected by a3 KD, neither under growth (Figure 2E) nor starvation conditions (Figure S2A). Forskolin treatment did indeed elicit redistribution of B2 positive vesicles from mostly perinuclear to more widely distributed throughout the cells, but failed to induce detectable plasma membrane insertion (Figure 2E).

We next asked if the V-ATPase, and specifically a3, is important for pH_i_ regulation in PDAC cells. BxPC3 cells were treated with forskolin, and recovery of pH_i_ after an NH_4_Cl prepulse-induced intracellular acid load determined. To isolate the V-ATPase contribution from that of HCO_3_^−^- and Na^+^-dependent transporters, recovery was quantified in absence of these ions. Under these conditions, pH_i_ recovery was absent, both in mock- and a3 KD cells (Figure 2F–I). Figure 2J summarizes these data as the net H^+^ efflux (J_H+_) in the absence of Na^+^ and as a function of pH_i_. Comparable data were obtained in Panc-1 cells, using two different siRNAs (n = 2 per condition, not shown). When Na^+^ was re-introduced, BxPC-3 cells recovered pH_i_ efficiently after the acid load (Figure 2F–I), consistent with the very prominent Na^+^/H^+^ activity in these cells [41].

These results show that a3 KD does not detectably alter V-ATPase localization in PDAC cells, nor does the V-ATPase contribute detectably to pH_i_ recovery after acidification.

### 3.3. V-ATPase Inhibition, but not a3 KD, Reduces Proliferation and Increases Cell Death

**Figure 3:**
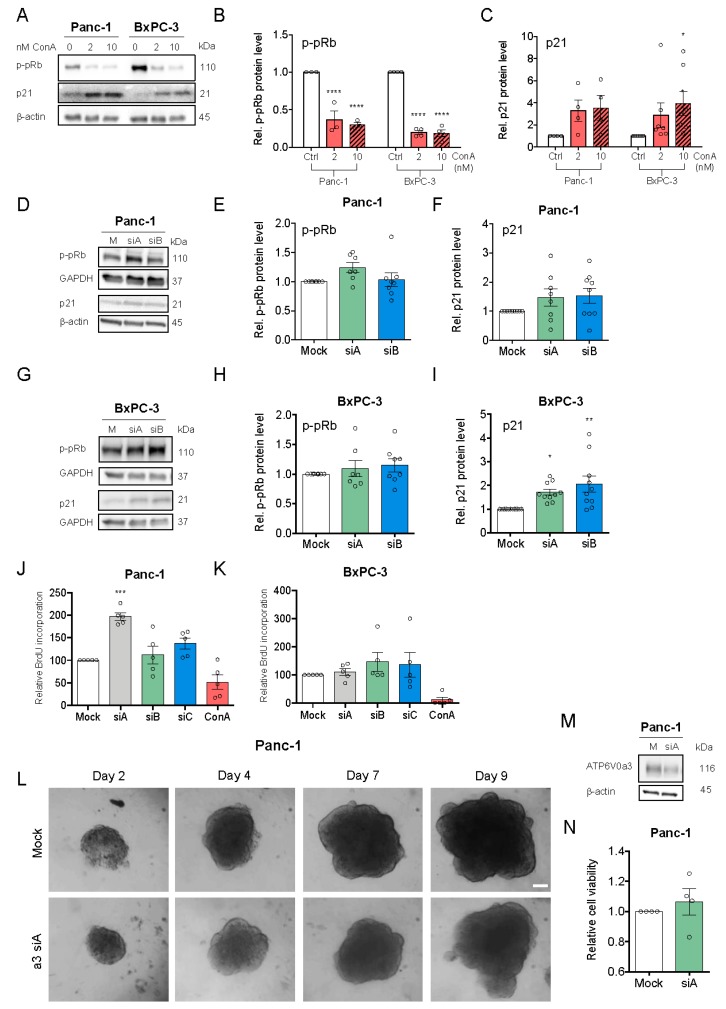
V-ATPase inhibition, but not a3 KD, reduces proliferation and increases cell death. (**A**–**C**) Panc-1 and BxPC-3 cells were treated with 2 or 10 nM ConA or vehicle ctrl. (DMSO) for 48 h and subjected to Western blotting for p-pRb and p21. (**A**) Representative blots. (**B**,**C**) Blot quantification. Data are normalized to β-actin (loading ctrl.) and the level in ctrl. conditions. Data is shown as mean with S.E.M. error bars, of 3 to 5 independent experiments per cell line. * *p* < 0.05 and **** *p* < 0.0001: Significantly different from the level in HPDE cells, using one-way ANOVA and Dunnett’s post hoc test. Rel. = relative. (**D**–**I**) Panc-1 (**D**–**F**) and BxPC-3 cells (**G**–**I**) were transfected with mock siRNA or 2 different siRNAs targeting a3, and grown for 48 h, lysed and subjected to Western blotting for p-pRb and p21. (**D**,**G**) Representative blots. (**E**,**F**,**H**,**I**) Quantification, normalized to GAPDH (loading ctrl.) and the level in the respective mock ctrl. Data is shown as mean with S.E.M. error bars, of 3 to 11 independent experiments per cell line and siRNA. * *p* < 0.05 and ** *p* < 0.01: Significantly different from the level in control conditions (mock siRNA), using one-way ANOVA and Dunnett’s post hoc test. Rel. = relative. (**J**,**K**) Panc-1 and BxPC-3 cells were transfected with mock siRNA or 3 different siRNAs targeting a3 or treated with 10 nM ConA, followed by BrdU analysis. Data are shown as mean with S.E.M. error bars, of 5 independent experiments per cell line. *** *p* < 0.001: Significantly different from the level in mock, using one-way ANOVA and Dunnett’s post hoc test. Rel. = relative. (**L**) Panc-1 cells were transfected with 2 different a3 siRNAs followed by seeding as 3D spheroids 48 h after transfection. (**L**) Representative images of Panc-1 spheroids after 9 days of growth. Scalebar = 100 μm. After 9 days, Panc-1 spheroids were subjected to Western blot analysis of a3 protein level to evaluate KD of a3 as compared to mock ctrl. (n = 3) (**M**) or to CellTiter Glo assay to measure cell viability as normalized to mock ctrl. (n = 4) (**N**).

To investigate the roles of the V-ATPase and specifically a3 in proliferation and stress signaling in PDAC cells, we determined the effect of the V-ATPase inhibitor ConA on levels of hyperphosphorylated retinoblastoma protein (p-pRb, proliferation marker) and p21 (Figure 3A–C). The p-pRb level was dose-dependently reduced by ConA, yet was conversely slightly increased by a3 KD (Figure 3D,E,G,H). Accordingly, BrdU incorporation was reduced by ConA treatment yet increased by a3 KD (Figure 3J–K). Both V-ATPase inhibition and a3 KD increased p21 expression (Figure 3A,C,D–I), with no effect on p-p53 (Figure S3F–H), and ConA treatment, yet not a3 KD, increased PARP cleavage (Figure S3A–E). To determine the possible PDAC cell dependence on a3 in a 3D setting more similar to the tumor microenvironment, 3D spheroids were generated from Panc-1 cells 48 h after a3 KD and spheroid growth monitored for 9 days, followed by viability quantification. Also under these conditions, a3 KD did not reduce growth or viability (Figure 3L–N). Similar data were obtained in BxPC-3 cells (Figure S3I–K).

These results show that V-ATPase inhibition strongly reduces proliferation and induces cell death in PDAC cells, while a3 KD slightly increases proliferation and does not elicit cell death. 

### 3.4. V-ATPase Inhibition Increases HIF-1α Protein Level, AMPK Activity and Alters Autophagic Flux

**Figure 4:**
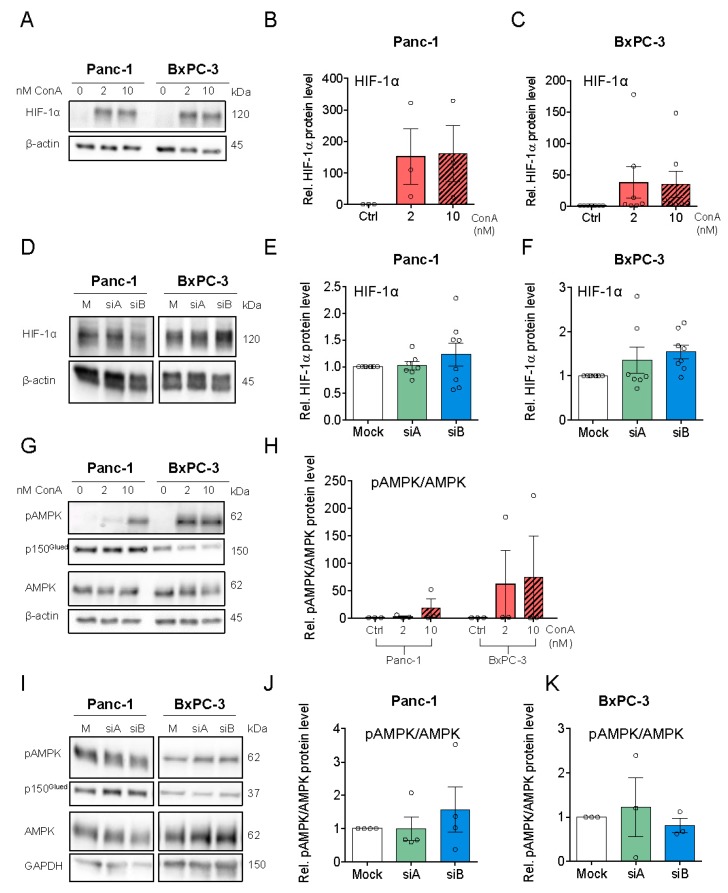
V-ATPase inhibition increases HIF-1α stabilization and AMPK activity and a similar trend is seen after a3 KD. Panc-1 and BxPC-3 cells were treated with either DMSO ctrl., 2 or 10 nM ConA for 48 h (**A**–**C**,**G**,**H**), or were transfected with Mock siRNA or 2 different siRNAs targeting a3, and grown for 48h (**D**–**F**,**I**–**K**), followed by Western blot analysis. (**A**,**G** and **D**,**I**) shows representative blots of HIF-1α, AMPK, and pAMPK in cells treated with ConA (n = 3–6) or in cells transfected with a3 siRNAs (n = 3–8). **B**,**E**,**F**,**H**,**J**,**K**) show quantification of the protein levels of HIF-1α (**B**,**C**,**E**,**F**) and pAMPK/AMPK (**G**–**K**) in cells treated with ConA or siRNA targeting a3. Data was normalized to beta-actin or GAPDH (loading ctrl.) and the level in respective DMSO or Mock ctrl. Data is shown as mean with S.E.M. error bars, of 3–6 independent experiments per cell line and siRNA. Rel. = relative.

Consistent with previous work [42], ConA treatment strongly increased HIF-1α protein in both Panc-1 and BxPC3 cells (Figure 4A–C), while only a slight trend in the same direction was seen upon a3 KD (Figure 4D–F). Given the major role of HIF-1α as a metabolic regulator, we reasoned that V-ATPase inhibition might elicit metabolic stress. In congruence with this notion, treatment with the V-ATPase inhibitor ConA, but not a3 KD, increased the level of phosphorylated, active AMP kinase (AMPK) (Figure 4G–K).

**Figure 5:**
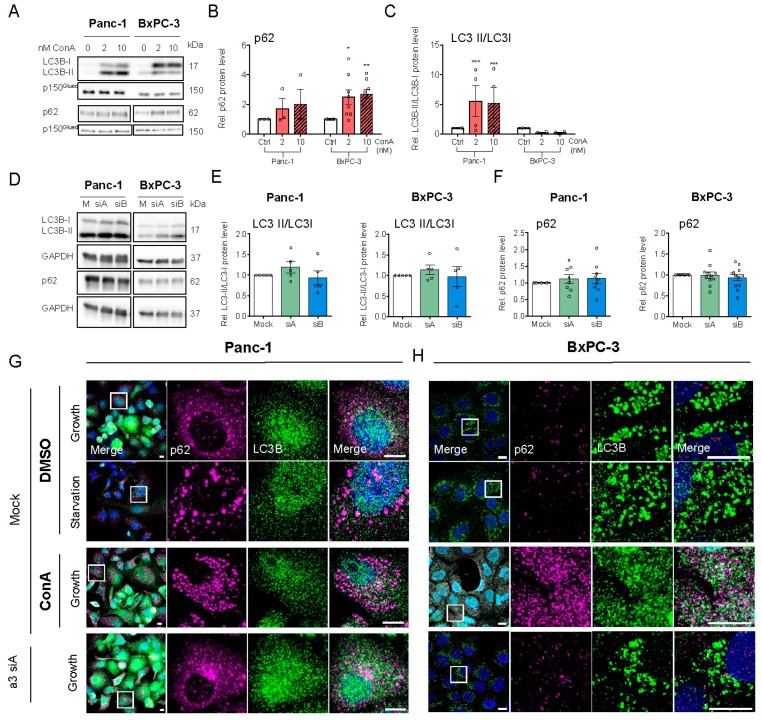
V-ATPase inhibition alters autophagic flux and a similar trend is seen after a3 KD. Panc-1 and BxPC-3 cells were treated with either DMSO ctrl., 2 or 10 nM ConA for 48 h (**A**–**C**), or were transfected with Mock siRNA or 2 different siRNAs targeting a3, and grown for 48 h (**D**–**F**), followed by lysing and Western blot analysis. (**A**,**D**) shows representative blots of LC3B and p62 in cells either treated with ConA (n = 3–7) (**A**) or transfected with siRNA against a3 (n = 3–12) (**D**). (**B**,**C**,**E**,**F**) show densitometric quantification of the protein levels of p62 (**B**,**F**) and LC3II/LCI (**C**,**E**). Data were normalized to respective loading ctrl. (p150^Glued^ or GAPDH) and the level in respective DMSO or mock ctrl. Data are shown as mean with S.E.M. error bars, of 3 to 12 independent experiments per cell line. * *p* < 0.05, ** *p* < 0.01, and *** *p* < 0.001: Significantly different from the level in control conditions (mock siRNA), using one-way ANOVA and Dunnett’s post hoc test. Rel. = relative. (**G**,**H**) Panc-1 and BxPC-3 cells were transfected with mock siRNA or siRNA targeting a3 and were simultaneously treated with either DMSO or 10 nM ConA, and were subjected to either growth or starvation conditions for 48 h. The cells were subsequently subjected to immunofluorescence analysis of LC3B (green) and p62 (magenta). Nuclei were stained with DAPI. Scalebar = 10 µm. Images represent 4 (Panc-1) and 5 (BxPC-3) independent experiments.

An increase in AMPK activity signals energy stress and induces upregulation of autophagy [43]. We therefore assessed the impact of ConA and a3 KD on autophagic markers and compared it to that of starvation, a known inducer of autophagy. A shift of LC3B-I to its PE-conjugated form, LC3B-II, and a shift from diffuse cytosolic to punctate staining of LC3B and the cargo receptor p62/SQSTM1 (p62) are indicative of increased autophagy, whereas increased accumulation of both LC3 and p62 indicates stalling of autophagy [44,45,46]. As seen from the representative blots, and consistent with the known ability of ConA and other V-ATPase inhibitors to stall autophagic flux [47], ConA treatment increased total LC3B, mainly as LC3B-II in Panc-1 cells and LC3B-I in BxPC-3 cells, and increased p62 in both cell lines (Figure 5A–C). A requirement for V-ATPase activity for LC3 lipidation, as seen in BxPC-3 cells, is a hallmark of unconventional autophagy [48], and this finding seems consistent with a previous report of differential regulation of LC3 lipidation in BxPC-3 and Panc-1 cells [49]. a3 KD modestly increased LC3B-II/LC3B-I in both cell types (Figure 5D,E), with little if any change in p62 (Figure 5D,F). In Panc-1 cells, glucose starvation, as well as ConA treatment, elicited p62 aggregation, and a3 KD markedly increased the colocalization of p62 and LC3B (Figure 5G). In BxPC-3 cells, ConA-induced p62 puncta were much smaller than those seen in Panc-1 cells, and starvation and a3 KD had no detectable effect on p62 and LC3B localization (Figure 5H).

Collectively, these results confirm the major impact of V-ATPase inhibition on the autophagic machinery of PDAC cells and indicate the role of a3 in this process in Panc-1 cells, albeit in absence of significant changes in the LC3B-II/-I ratio and p62 level.

### 3.5. a3 Negatively Regulates Migration and Invasion of PDAC Cells

**Figure 6:**
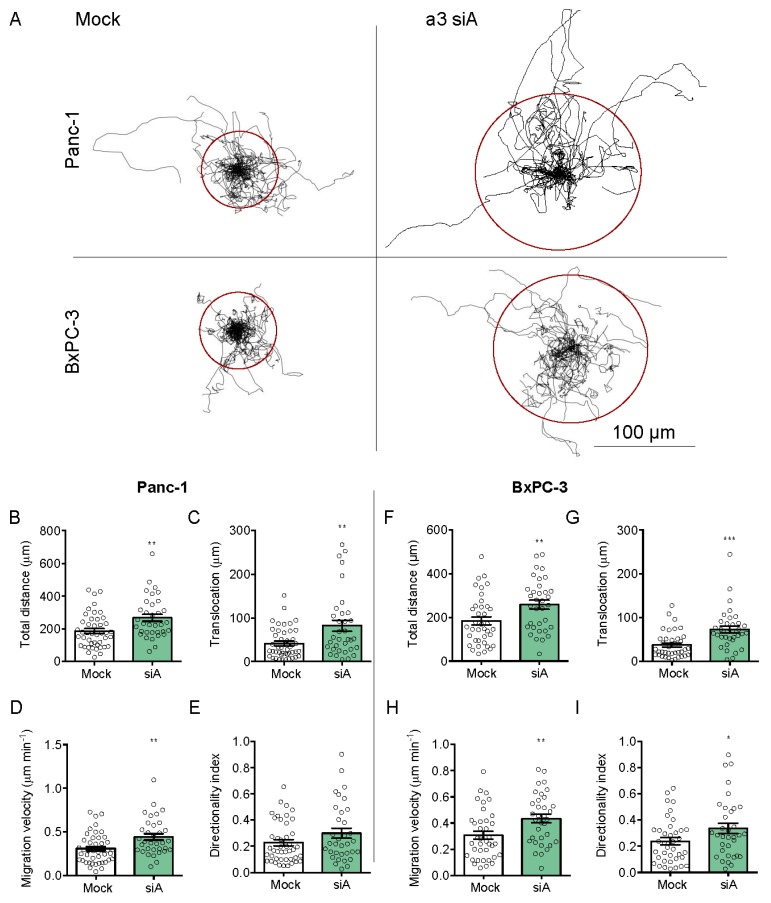
a3 negatively regulates the 2D migration of PDAC cells. (**A**) Trajectories of migrating mock- and a3 siRNA-transfected Panc-1 and BxPC-3 cells. The trajectories obtained were standardized to the same starting post represented by the center of a circle. The radii of the circles represent the mean distances (=translocation) covered within 10 h. Scalebar = 100 μm. (**B**–**I**) Total distance covered, translocation, velocity, and directionality index, calculated based on all data as in (**A**). Data are representative of 4 independent experiments (44 mock and 34 a3 siRNA cells) for Panc-1 cells and 3 experiments (38 mock and 34 a3 siRNA cells) for BxPC-3 cells. Data are shown as mean with S.E.M. error bars. * *p* < 0.05, ** *p* < 0.01, and *** *p* < 0.001: Significantly different from the level in control conditions (mock siRNA), using Student’s *t*-test.

To our knowledge, there are no published reports of the effect of a3 KD on PDAC cell migration. Panc-1 and BxPC-3 cell migration were assessed on a matrix simulating the composition of PDAC ECM (collagen I, laminins, fibronectin, collagen III, and collagen IV [50]). Mock- and a3 KD cells were seeded sparsely and single cell migration analyzed as total distance migrated, directionality index, migration velocity, and translocation (see Materials and Methods). Figure 6A shows trajectories of single migrating cells, normalized to a common starting point (see Videos S1–S4), and Figure 6B–I show the summarized data. Strikingly, a3 KD significantly increased all four parameters in BxPC-3 cells, and all except directionality in Panc-1 cells. Similar results were obtained when cell migration was measured on matrigel (Figure S4A–H). Thus, a3 KD significantly increased migration in both cell lines and on two different matrices.

**Figure 7:**
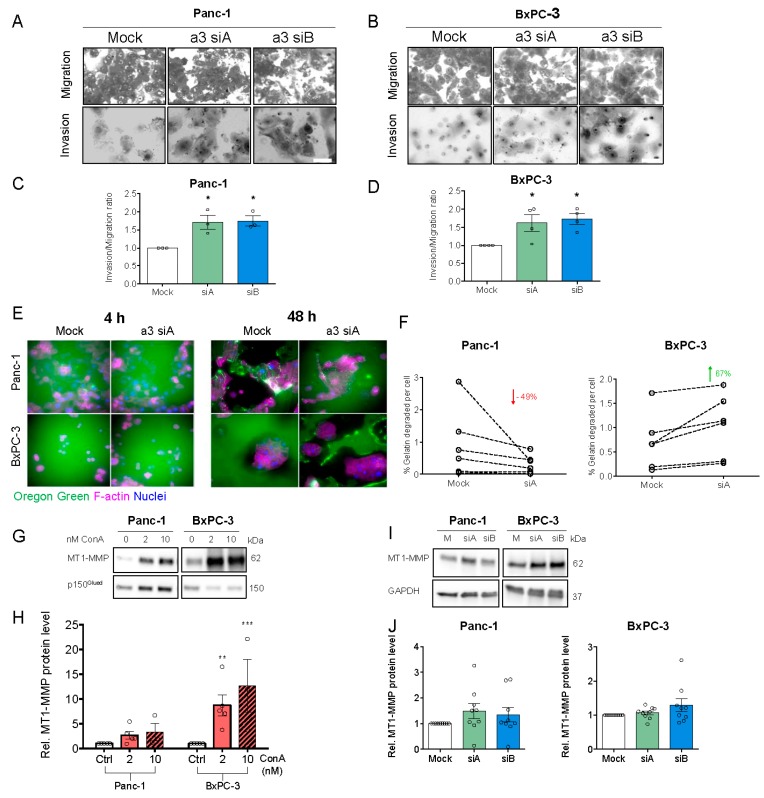
a3 negatively regulates the invasion of PDAC cells through matrigel. (**A**,**B**) Panc-1 or BxPC-3 cells were transfected with mock siRNA or 2 different siRNAs targeting a3 prior to seeding onto matrigel invasion chambers. Experiments were terminated after another 22 to 24 h, as described in Materials and Methods. Upper panels (**A**,**B**) show representative images of cells adhering to the lower chamber stained with Giemsa solution after the invasive process. Scalebars = 40 µm. (**C**,**D**) show relative invasion, as determined by the number of cells invaded through matrigel-coated inserts. Data are shown as mean ± S.E.M. of 3 to 4 independent experiments per condition. * *p* < 0.05: Significantly different from the level in control conditions (mock siRNA), using one-way ANOVA and Dunnett’s post hoc test. (**E**) Panc-1 and BxPC-3 cells were transfected with mock siRNA or siRNA targeting a3 and grown for 48 h, followed by seeding onto Oregon-green conjugated gelatin-coated coverslips and growth for 4 or 48 h. The cells were subsequently subjected to immunofluorescence analysis of F-actin (Alexa-568-phalloidin (magenta), and Oregon-green gelatin). Nuclei were stained with DAPI. Scalebar = 40 µm. (**F**) Shows the quantification of gelatin degradation (performed in ImageJ) in Panc-1 and BxPC-3 cells after 48 h, normalized to the number of cells. Data represents 6 independent experiments per condition. A Student’s *t*-test was used to test for significant differences between mock and siA conditions. (**G–J**) Panc-1 and BxPC-3 cells were treated with DMSO (ctrl.), 2 or 10 nM ConA for 48 h (**G**–**H**) or were transfected with Mock siRNA or 2 different siRNAs targeting a3 and grown for 48 h (**I**,**J**), followed by Western blot analysis. (**G**,**I**) Representative blots of MT1-MMP and loading controls. (**H–J**) Quantification of the MT1-MMP protein level. Data was normalized to GAPDH or p150^Glued^ (loading ctrl.) and the level in respective mock growth or DMSO ctrl. cells. Data is shown as mean with S.E.M. error bars, of 3 to 5 independent experiments per cell line and treatment. * *p* < 0.05, ** *p* < 0.01, and *** *p* < 0.005: Significantly different from the level in control conditions (mock siRNA or DMSO ctrl.), using one-way ANOVA and Dunnett’s post hoc test. Rel. = relative.

In congruence with this, a3 KD significantly increased the invasion of both cell types through matrigel (Figure 7A–D). To understand the mechanisms through which a3 KD increased invasion, we assessed the roles of the V-ATPase and specifically a3 in the regulation of matrix degradation. After a3 KD, PDAC cells were seeded on Oregon-green gelatin-covered coverslips and the gelatin was evaluated after 4 and 48 h (Figure 7E,F). Interestingly, a3 KD decreased matrix degradation by Panc-1 cells by about 50%, yet increased it in BxPC-3 cells by about 67% (Figure 7F). V-ATPase activity was reported to be important for the degradation of MT1-MMP (also known as MMP14), which plays a key role in PDAC cell invasion [33] and is an activator of MMP2 and, via MMP2, also MMP9 [31,51]. In congruence with this, MT1-MMP expression was increased by ConA treatment (Figure 7G,H), yet not or only marginally by a3 KD (Figure 7I,J).

These results show that a3 negatively regulates migration and invasion of Panc-1 and BxPC-3 cells, yet the mechanisms may differ between the two cell lines and do not seem to involve MT1-MMP upregulation.

## 4. Discussion

PDAC is an exceptionally aggressive cancer type relying heavily on nutrient scavenging and autophagy, processes dependent on endo-/lysosomal V-ATPase activity [24,25,26]. It was previously reported that in some but not all PDAC cells, V-ATPases localize to the plasma membrane and their inhibition attenuates invasion [27]. However, in addition to these cell-type differences, including no net effect on Panc-1 cell invasion, conclusions regarding invasion were drawn based on scratch wound assays and migration into an agar disc, i.e., very far from the matrix conditions in PDAC tumors [27]. Here, we focused on the ATP6V0a3 (a3) subunit, which was proposed to target the V-ATPase to the plasma membrane in some invasive breast cancer and melanoma cells [20,21,22] and was previously detected in the plasma membrane of PDAC cells [27]. We found mRNA and protein levels of a3 but not of B2 to be increased in PDAC cell lines compared to immortalized human pancreatic duct epithelial cells. This suggests that a3 expression is increased in the PDAC cell lines studied, in the absence of total V-ATPase upregulation, in line with recent work in breast cancer cells [52].

Under control conditions, a3 mainly localized to LAMP1- and Rab7-positive vesicles in agreement with previous work [15,39], i.e., it was predominantly late endosomal/lysosomal. Although previous work proposed a3 localization to the plasma membrane in PDAC [27,29], the overall localization pattern appears similar to that found in the present study, with a marginal fraction of total a3 staining found in the plasma membrane. In agreement with our findings under control conditions in PDAC cells, little if any V-ATPase expression was detectable in the plasma membrane of melanocytes [12]. In contrast, the introduction of a wound scratch elicited significant redistribution of a3 to the plasma membrane in BxPC-3 cells, consistent with previous findings [23]. This agrees well with previous work showing a role of a3-Rab7 interactions in secretory lysosome trafficking in osteoclasts [39]. Interestingly, a3 membrane translocation did not happen in subconfluently seeded cells with no wound scratch, suggesting that it may be caused by the wounding of the cells, which releases motility-stimulating molecules such as ATP.

An increase in cAMP elicits V-ATPase membrane insertion in some cell types [40], yet in our hands, only a redistribution of a3 to more peripheral intracellular regions was detectable upon forskolin treatment of PDAC cells; a3 knockdown had no effect on pH_i_ recovery after acidification, in absence or presence of forskolin. Thus, even if a minor fraction of V-ATPases not detectable in our immunofluorescence analysis was present in the plasma membrane, this seems to be of little if any functional relevance for pH_i_ regulation under the conditions studied. While a small contribution of V-ATPase activity to pH_i_ regulation has been reported in intact pancreatic ducts [53], our data are consistent with pH_i_ recovery after an acid load being largely or fully accounted for by the activity of Na^+^/H^+^ exchangers and Na^+^-HCO_3_^−^ cotransporters in most cancer cells [54,55] and with recent work showing that depletion of V-ATPases did not alter submembranous pH in Panc-1 cells [15]. 

V-ATPases play important roles in nutrient response and proliferation [56,57,58]. ATPase inhibition decreased proliferation and increased p21 expression in PDAC cells, and increased HIF-1α protein level and AMPK activity, in congruence with previous work [59,60]. While the upregulation of HIF-1α by V-ATPase inhibition was previously reported, the proposed mechanisms differ between studies, likely reflecting both cell-type-, inhibitor- and dosage-dependent effects [59,60]. HIF-1α upregulation by bafilomycin A in PC3 prostate cancer cells was pH_i_ independent [60], in congruence with the lack of effect of a3 KD on pH_i_ in our study. In PC3 cells, HIF-1α upregulation by bafilomycin was found to reflect a bafilomycin-induced interaction of ATP6V0C with HIF-1α, resulting in reduced HIF-1α degradation [60]. HIF-1α undergoes not only proteasomal but also lysosomal degradation (specifically chaperone-mediated autophagy) [61]. Thus, HIF-1α upregulation by interfering with V-ATPase function may at least in part reflect its reduced lysosomal degradation. Finally, it was recently reported that the HIF-1α upregulation upon V-ATPase inhibition was downstream from disturbed cellular iron metabolism due to interference with transferrin receptor trafficking [62]. Similar to our findings, p21 was also upregulated in a HIF-1α-dependent but a p53-increase-independent manner by bafilomycin A in SiHa cervical cancer cells [60]. These two events may be linked, as in PC3 cells, HIF-1α was only marginally transcriptionally active towards its normal targets after bafilomycin-induced stabilization, yet elicited the upregulation of p21 by dissociating c-myc from the p21 repressor [60]. 

V-ATPase activity is essential for the maintenance of autophagic flux, and consistent with this, V-ATPase inhibition caused p62- and LC3B accumulation and a change in autophagic vesicle morphology in both cell types. a3 KD had no detectable effect on p62, and only caused a slight increase in the LC3B-II/LC3B-I ratio. However, under starved conditions, a marked increase in p62/LC3 colocalization was seen after a3 KD in Panc-1 cells, indicative of reduced autophagic flux. This is in congruence with the notion that the V0 domain is important for the docking and fusion between secretory vesicles, although the role of a3 in this process is subject to controversy [39,63]. Whether the role of a3/V-ATPase in cell death/proliferation signaling is dependent on altered H^+^ pumping by the V-ATPase remains unresolved. Clearly, however, not all functions of neither a3 nor the V-ATPase per se are dependent on H^+^-transport [6,64]. 

Contrary to our expectation based on a series of papers on this topic in breast cancer [18,19,21], a3 KD significantly increased both 2D migration and invasion. This finding was consistent between Panc-1 and BxPC-3 cells and on two different migration matrices. In conjunction with the lack of detectable a3 plasma membrane localization under control conditions in these cell lines [21], this strongly suggests that the role of a3 in PDAC cell invasiveness differs from that proposed in melanoma and breast cancer cells [18,20,21,22]. A major difference between this and previous work is that in Panc-1 and BxPC-3 cells, a4 was undetectable using qPCR analysis, consistent with in silico analyses suggesting minimal if any a4 expression. It is notable that also in MDA-MB-231 cells, KD of a4, but not of a3, reduced plasma membrane V-ATPase expression and a4 KD inhibited invasion more than did a3 KD [21]. Furthermore, in 4T1-12B breast cancer cells, CRISPR/Cas9 knockout of a3 had no effect on migration, invasion, and plasma membrane V-ATPase localization, whereas all of these parameters were inhibited by a4 knockout [23]. We therefore conclude that the evidence for a role of a3 in plasma membrane targeting and invasion of cancer cells is limited to a few cell types, and at least in some cases seems linked to the expression of a4. 

Consistent with previous reports [33,65], MT1-MMP activity was upregulated by V-ATPase inhibition. However, MT1-MMP activity was at most marginally affected by a3 KD and thus seems unlikely to account for the stimulation of migration and invasion by a3 KD. Conversely, MT1-MMP knockdown reverted a proposed stimulatory effect of ConA on autophagy in glioblastoma cells [66]. This possibility was not further addressed here, given that in our hands, ConA blocks autophagic flux, consistent with the generally reported effect of this and other V-ATPase inhibitors [47]. The stimulatory effect of a3 KD on migration was similar on matrigel and a complex matrix corresponding closely to the PDAC tumor microenvironment [50]. Consistent with this, ECM degradation was increased upon a3 KD in BxPC-3 cells. However, conversely, ECM degradation was inhibited by a3 KD in Panc-1 cells. BxPC-3 cells are KRAS wild type and harbor a homozygous deletion of SMAD4, while Panc-1 cells have a constitutively active KRAS mutation and are SMAD4 wild type [37]. Given the known roles of both mutations in regulating epithelial-to-mesenchymal transition and invasive behavior in PDAC [67,68], this may play a role in the observed difference between the cell types; however, this remains to be addressed. 

Thus, further studies are required to determine the mechanisms involved in the unexpected stimulation of migration and invasion by a3 KD. a3 has been assigned important roles in both plasma membrane cholesterol localization in PDAC cells [15] and secretory lysosome fusion in osteoclasts [39], and involvement of both of these processes is possible and should be addressed in future work. We speculate that the stimulation of migration and invasion by a3 knockdown demonstrated in this work is likely related to roles in cell motility of V-ATPase dependent processes such as many cellular signaling pathways and endo-lysosomal trafficking [6,7,14,16]. Importantly, our work confirmed that a3 is upregulated in PDAC cells, and given the complexity of the in vivo microenvironment of PDAC tumors, it remains possible that a3 is a relevant target in anticancer therapy in some PDAC types or conditions. The involvement of the V-ATPases per se, and the a subunits, in particular, in cancer, are still incompletely understood. This is exemplified by the striking yet unexplained differences between the roles of different a subunits in invasion/migration in different cancers [18,20,21,22,23], but the roles of a3-containing V-ATPases in other cancer hallmarks, such as chemotherapy resistance [7] and macropinocytosis [15], should be explored in future studies. 

In conclusion, the a3 subunit of the V-ATPase was upregulated in PDAC cells compared to non-cancer pancreatic epithelial cells. Under unstimulated conditions, a3 localization was mainly endo-/lysosomal, and its knockdown had no detectable effect on pH_i_ regulation after acid loading. V-ATPase inhibition increased HIF-1α expression and decreased proliferation and autophagic flux under both starved and non-starved conditions, whereas a3 KD had little or no effect on these parameters. Also, spheroid growth of PDAC cells was unaffected by a3 KD. Remarkably, a3 KD increased migration and invasion of Panc-1 and BxPC-3 PDAC cells, and increased gelatin degradation in BxPC-3 cells, yet decreased it in Panc-1 cells. We conclude that in these PDAC cell lines, a3 negatively regulates migration and invasion and that a3-dependence of extracellular matrix degradation likely contributes to, but cannot fully account for, this effect.

## Supplementary Materials

The following are available online at https://zenodo.org/record/3696682#.XmDDrfSYOpp, Figure S1: (A,D) Panc-1 cells subjected to growth, starvation, subconfluent or wound scratch conditions were subjected to immunofluorescence analysis for a3 (green) and LAMP1 or Cortactin (magenta). Nuclei were stained with DAPI. Scalebar = 10 µm. (B,C) Panc-1 or BxPC-3 cells subjected to growth conditions were transfected with empty vector (ctrl.) or a3-GFP and LAMP1-mCherry vector and subjected to immunofluorescence analysis for LAMP1 (magenta). Nuclei were stained with DAPI. Scalebar =10 µm. (E,F) Subconfluent Panc-1 or BxPC-3 cells were subjected to immunofluorescence analysis for a3 (green) and Golgin-97, Rab7 or LAMP1 (all magenta). Nuclei were stained with DAPI. Scalebar = 10 µm. Images are representative of 2–5 independent experiments, Figure S2: (A) Panc-1 or BxPC-3 cells were transfected with Mock siRNA or siRNA targeting a3, and were grown under starvation conditions for 48 h, in presence of either 10 M DMSO or Forskolin, prior to immunofluorescence analysis, using anti-B2 antibody (green) in conjunction with anti-E-cadherin (magenta). Nuclei were stained with DAPI. Scalebar = 20 µm B-I) Relative mRNA levels of ATP6V0a1, ATP6V0a2, ATP6V0a3 and ATP6V1B2 in siRNA-transfected Panc-1 and BxPC-3 cells were assessed by qRT-PCR. Data was normalized to the mRNA level of beta-actin and the level in respective Mock siRNA ctrls. Data is shown as mean with S.E.M. error bars, of three independent experiments per cell line. One-Way ANOVA and Dunnett’s post hoc test was used to test for significant differences between Mock siRNA and a3 siRNA. Rel. = relative, Figure S3: Panc-1 and BxPC-3 cells were treated with either DMSO ctrl, 2 or 10 nM ConA for 48 h (A,B), or were transfected with Mock siRNA or 2 different siRNAs targeting a3, and grown for 48 h (C–H), followed by lysing and Western blot analysis of cPARP/PARP (A–E) and p-p53 (F–H). (A,C,F) show representative blots of cPARP/PARP (A,C) and p-p53 (F) and (B,D,E,G,H) show densitometric quantification normalized to respective loading ctrls (β-actin, GAPDH or p150Glued) and the level in either Mock ctrl or DMSO ctrl cells. Data is shown as mean with S.E.M. error bars, of 3–5 independent experiments per cell line. Rel. = relative. (I) BxPC-3 cells were transfected with Mock siRNA or siRNAs targeting a3 followed by seeding as 3D spheroids 48 h after transfection. (I) shows representative images of Panc-1 spheroids after 9 days of growth. Scalebar = 100 µm. After 9 days, BxPC-3 spheroids were subjected to Western blot analysis of a3 protein level to evaluate KD of a3 as compared to mock ctrl (n = 3) (J). BxPC-3 spheroids were subjected to CellTiter Glo assay to measure cell viability as normalized to mock ctrl (n = 5) (K). A Student’s *t*-test was used to test for significant differences between Mock siRNA and a3 siRNA, Figure S4: Migration of mock- and a3 siRNA-transfected Panc-1 BxPC-3 cells on Matrigel. (A–G) Total distance covered, translocation, velocity, and directionality index, calculated based on all trajectory data (not shown). Data are representative of 3 independent experiments (27 mock and 34 a3 siRNA cells) for Panc-1 cells and 3 experiments (42 mock and 33 a3 siRNA cells) for BxPC-3 cells. Data given as means with S.E.M. error bars. ** (*p* < 0.01), *** (*p* < 0.001), and **** (*p* < 0.0001): Significantly different from the level in control conditions (mock siRNA), Student’s *t*-test, Video S1: Time-lapse imaging of migrating Panc-1 cells transfected with mock siRNA, Video S2: Time-lapse imaging of migrating Panc-1 cells transfected with a3 siRNA, Video S3: Time-lapse imaging of migrating BxPC-3 cells transfected with mock siRNA, Video S4: Time-lapse imaging of migrating BxPC-3 cells transfected with a3 siRNA.
